# TrypTag.org: A Trypanosome Genome-wide Protein Localisation Resource

**DOI:** 10.1016/j.pt.2016.10.009

**Published:** 2017-02

**Authors:** Samuel Dean, Jack D. Sunter, Richard J. Wheeler

**Affiliations:** 1 Sir William Dunn School of Pathology, University of Oxford, Oxford, UK

TrypTag is a major resource which will contain the localisation of every protein encoded in the *Trypanosoma brucei* genome. Localisations of over 2000 proteins are already available *via* http://tryptag.org. This will be a transformative resource for enabling sophisticated analysis of conserved eukaryotic and parasite specific cell biology.

TrypTag is a new community resource with the aim of providing the localisation of every protein encoded in the trypanosome genome. This resource grew out of projects in the Gull Lab where we needed to determine hundreds of protein localisations using fluorescent protein tagging to validate proteomic analyses 1, 2. Endogenous tagging of protein genes using long-primer PCR to generate the construct was developed for *Trypanosoma brucei* several years ago 3, 4, 5. This methodology was optimised to make it efficient, scalable, and reproducible [6], and was updated to a high-throughput 96-well plate format [7], allowing us to generate and image up to 192 procyclic form (tsetse lifecycle stage) cell lines each week and make them available online (Figure 1). TrypTag is funded by the Wellcome Trust as a community resource.

Trypanosomes, including *T. brucei*, have an exquisitely structured shape with a precisely defined and polarised architecture. Many major organelles (including the mitochondrion, flagellum, and Golgi apparatus) are present in a single copy at a consistent location, and divide through a precise series of morphogenetic events. This means that, in trypanosomes, protein localisation indicates precisely which organelle a protein is associated with, and can be extremely informative for function (whilst recognising the caveats such as the fact that these are mutant forms of the protein, that cellular addressing signals may be ablated, and that presence in a location may be functionally inconsequential). Forty percent of *T. brucei* protein-coding genes are annotated as hypothetical with minimal functional information, and analysis of individual proteins has lagged behind scaled highly parallel technologies like RNAseq, mass spectrometry, etc.

Understanding *T. brucei* biology is informative for two main reasons: these parasites are among the most divergent eukaryotes; conserved structures in *T. brucei* therefore represent eukaryote-wide core biology. Non-conserved structures represent divergent biology, candidates for understanding unique important features for pathogenicity.

Once complete, the resource will include localisation data for all protein-coding genes, excluding variant surface glycoprotein (VSG) genes (the surface protein for antigenic variation) and genes not assigned to a chromosome. Whether a gene is scheduled to be tagged can be viewed on TrypTag.org. The resource is currently based on *T. brucei* TREU927 genome version 5.1 (gene models from August 2013), but will incorporate new genome releases and published gene model changes. Proteins with an annotated signal peptide have been tagged at only the C terminus; all other proteins will be tagged at the N and C termini. No other signal sequences or post-translational modifications (e.g., glycophosphatidylinositol anchor) have been used as criteria to exclude tagging of a terminus. This gave a final set of 7168 and 8129 cell lines to generate, respectively (15 297 total). Given our current throughput, we expect this resource to be complete in 2019.

The TrypTag resource comprises image data of more than 200 cells per cell line (including all stages of the cell cycle and rare cell cycle events) and human interpretation and annotation of protein localisation and cellular structures (Figure 1). Cell lines are not stored as it is essentially as quick to remake a cell line as to freeze and thaw a stabilate, and the speed and flexibility of the long-primer PCR system allows addition of a different tag (e.g., epitope, alternative fluorescent protein, electron microscopy) by changing only the plasmid template. Localisation annotations are drawn from a hierarchical ontology designed specifically for trypanosomes, each linked with a cellular component gene ontology (GO) term. Additionally, localisation modifiers are used for describing localisation of tagged proteins to subdomains of organelles or when unknown, but specific patterns are observed (Figure 2).

The data are accessible as a community resource in three ways. Firstly, an example field of cells for each cell line (both N and C terminally tagged) is shown on the TrypTag website, and includes annotation and primer sequence information. Secondly, example cells will be shown on TriTrypDB [8] gene pages, released with their periodic updates, searchable via GO terms, and linked to the full TrypTag database. Thirdly, the raw entire dataset will ultimately be available via an FTP server and will be comprised of open microscopy environment (OME) Tiff images and a javascript short object notation (json) metadata file that describes the localisation, GO terms, and tagging primer sequences for a protein (Figure 1).

The data for the 2000 protein localisations available now at http://tryptag.org were announced at the BSP Trypanosomiasis and Leishmaniasis Seminar 2016 (České Budějovice, Czech Republic). Phased additions of data will appear regularly. We anticipate that TrypTag will be a transformative resource for the community. It will allow researchers to rapidly identify or validate localisations of proteins of interest. More importantly, this resource will enable fundamentally new types of analysis. The analysis of large numbers or cohorts of proteins will allow, for example, identification of new signals that target proteins to cellular structures, signals responsible for cell-cycle-dependent localisation and stability, and organelle subcompartments using protein and gene sequence motif analysis tools. Definition of localisation profiles of gene families (e.g., kinases) and proteins that have been post-translationally modified (e.g., palmitoylated) will allow determination of their function in spatial terms. Enabling researchers to move from organelle-based proteomics will stimulate development of more advanced analyses such as interactomics. The proteins identified by TrypTag will enable the generation of reporter cell lines for sophisticated functional assays. We are keen to collaborate with the community for these types of analyses. However, we are happy for researchers wanting to use the localisation data for one or two proteins to simply cite the TrypTag website and this publication.

## Figures and Tables

**Figure 1:**
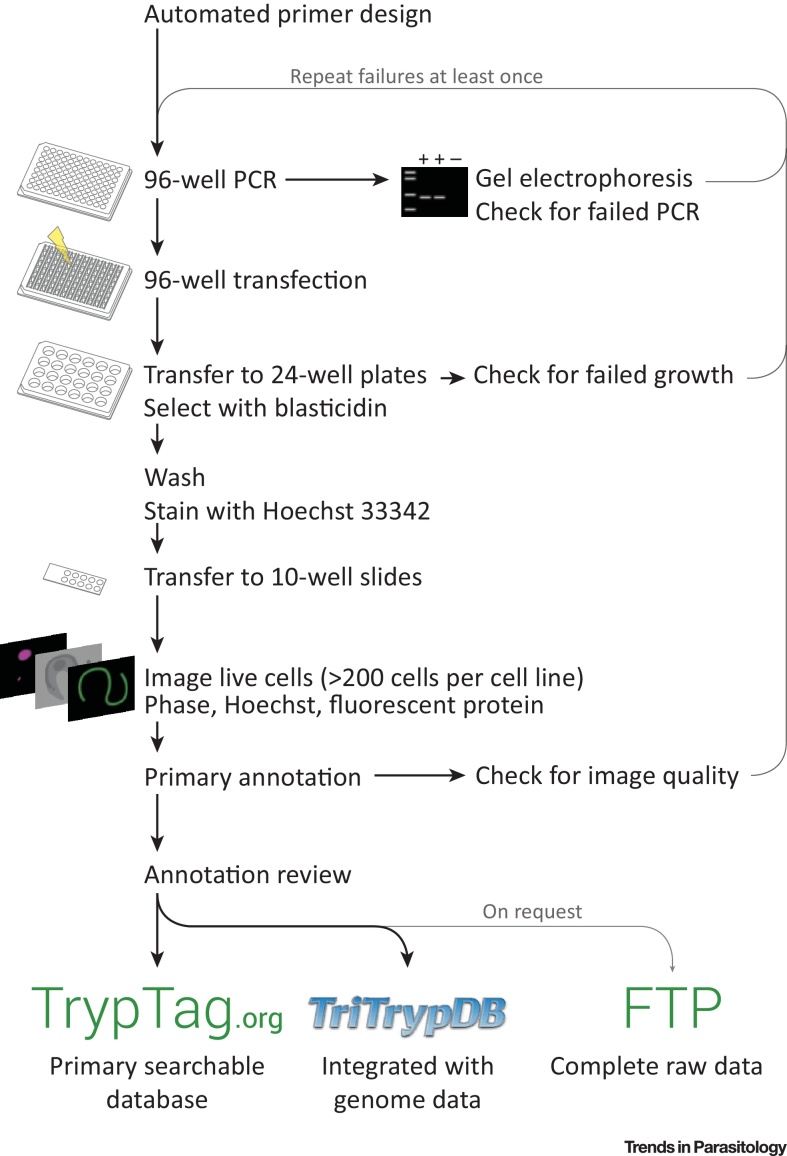
High-Throughput Protein Tagging Workflow. Schematic of the tagging workflow from automated primer design to data release routes.

**Figure 2:**
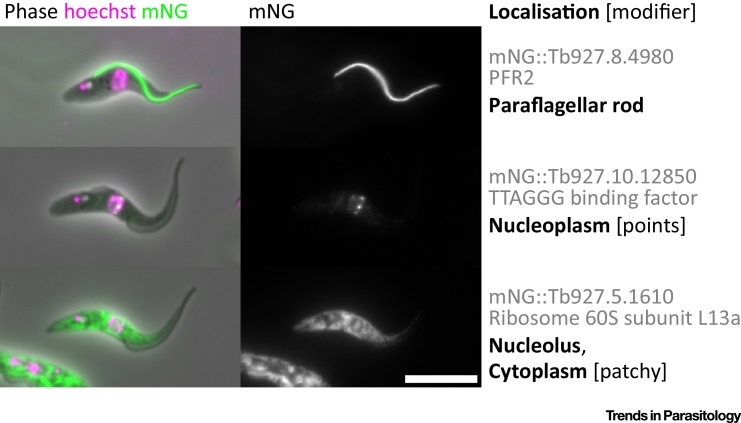
Example Localisations and Annotations from TryTag.org. Three example cell lines expressing proteins tagged at the N terminus with mNeonGreen (mNG) [9], and imaged along with phase contrast (phase) and Hoechst 33342, a fluorescent marker for DNA in the nucleus and kinetoplast (hoechst). Localisation annotations are assigned in the form localisation 1 [modifier 1, modifier 2, …], localisation 2 [modifier 1, …], …. Each localisation has a linked cellular component gene ontology (GO) term. For example, in the middle, Tb927.10.12850, tagged on its N-terminus by mNG, localises to the nucleoplasm (GO:0005654). However, it does not localise to the entire nucleoplasm, so its localisation is further defined by the modifier term ‘points’. Scale bar represents 10 μm.

## References

[1] Dean S. (2016). Cilium transition zone proteome reveals compartmentalization and differential dynamics of ciliopathy complexes. Proc. Natl. Acad. Sci. U. S. A..

[2] Sunter J.D. (2015). A dynamic coordination of flagellum and cytoplasmic cytoskeleton assembly specifies cell morphogenesis in trypanosomes. J. Cell Sci..

[3] Arhin G.K. (2004). A PCR-based method for gene deletion and protein tagging in *Trypanosoma brucei*. Methods Mol. Biol..

[4] Gaud A. (1997). Polymerase chain reaction-based gene disruption in *Trypanosoma brucei*. Mol. Biochem. Parasitol..

[5] Oberholzer M. (2006). A vector series for rapid PCR-mediated C-terminal *in situ* tagging of *Trypanosoma brucei* genes. Mol. Biochem. Parasitol..

[6] Dean S. (2015). A toolkit enabling efficient, scalable and reproducible gene tagging in trypanosomatids. Open Biol..

[7] Dyer P. (2016). High-throughput gene tagging in *Trypanosoma brucei*. J. Vis. Exp..

[8] Aslett M. (2010). TriTrypDB: a functional genomic resource for the Trypanosomatidae. Nucleic Acids Res..

[9] Shaner N.C. (2013). A bright monomeric green fluorescent protein derived from *Branchiostoma lanceolatum*. Nat. Methods.

